# Using tropical reef, bird and unrelated sounds for superior transfer learning in marine bioacoustics

**DOI:** 10.1098/rstb.2024.0280

**Published:** 2025-06-12

**Authors:** Ben Williams, Bart van Merriënboer, Vincent Dumoulin, Jenny Hamer, Abram B. Fleishman, Matthew McKown, Jill Munger, Aaron N. Rice, Ashlee Lillis, Clemency White, Catherine Hobbs, Tries Razak, David Curnick, Kate E. Jones, Tom Denton

**Affiliations:** ^1^ University College London, London, UK; ^2^ Institute of Zoology, Zoological Society of London, London, UK; ^3^ DeepMind, Google Inc, Mountain View, CA, USA; ^4^ DeepMind, Google Inc, Canada; ^5^ DeepMind, Google Inc, New York, USA; ^6^ Conservation Metrics, Santa Cruz, CA, USA; ^7^ University of New Hampshire, Durham, NH, USA; ^8^ Bioacoustics Research Program, Cornell University, Ithaca, NY, USA; ^9^ Sound Ocean Science, Gqeberha, Eastern Cape, South Africa; ^10^ University of Bristol, Bristol, UK; ^11^ University of Exeter, Exeter, UK; ^12^ IPB University, Bogor, Jawa Barat, Indonesia

**Keywords:** bioacoustics, passive acoustic monitoring, soundscape, machine learning, deep learning, coral reef, marine

## Abstract

Machine learning has the potential to revolutionize passive acoustic monitoring (PAM) for ecological assessments. However, high annotation and computing costs limit the field’s adoption. Generalizable pretrained networks can overcome these costs, but high-quality pretraining requires vast annotated libraries, limiting their current development to data-rich bird taxa. Here, we identify the optimum pretraining strategy for data-deficient domains, using tropical reefs as a representative case study. We assembled ReefSet, an annotated library of 57 000 reef sounds taken across 16 datasets, though still modest in scale compared to annotated bird libraries. We performed multiple pretraining experiments and found that pretraining on a library of bird audio 50 times the size of ReefSet provides notably superior generalizability on held-out reef datasets, with a mean area under the receiver operating characteristic curve (AUC-ROC) of 0.881 (±0.11), compared to pretraining on ReefSet itself or unrelated audio, with a mean AUC-ROC of 0.724 (±0.05) and 0.834 (±0.05), respectively. However, our key findings show that cross-domain mixing, where bird, reef and unrelated audio are combined during pretraining, provides superior transfer learning performance, with an AUC-ROC of 0.933 (±0.02). SurfPerch, our optimum pretrained network, provides a strong foundation for automated analysis of tropical reef and related PAM data with minimal annotation and computing costs.

This article is part of the theme issue ‘Acoustic monitoring for tropical ecology and conservation’.

## 1. Introduction

Advanced monitoring tools are key to tackling the biodiversity crisis [1]. Passive acoustic monitoring (PAM) represents a powerful medium through which to gather data for ecological assessments [2,3]. Low-cost autonomous recording units are now widely available, enabling the collection of vast quantities of PAM data with considerably lower logistical and expertise costs in the field [4–6]. However, a boom in their accessibility and application has resulted in data collection scaling beyond the analytical capacity of human annotators [2]. Effective automated analysis is therefore required to alleviate this analytical bottleneck and maximize the potential of these data. Machine learning (ML) has emerged as a powerful tool with the potential to meet this demand, with state-of-the-art approaches typically using deep neural networks [7]. The current paradigm for ML-accelerated PAM analysis employs supervised learning techniques to train classifiers that can detect target signals. These supervised learning techniques are typically used to develop species-level detectors or identify anthropogenic activities [2,7].

A key drawback of traditional supervised approaches is their reliance on large annotated libraries of validated target sounds, typically requiring hundreds of examples per class to train an accurate classifier. These libraries are costly and time-consuming to assemble, primarily owing to their reliance on human annotators [8]. Additionally, classifiers often generalize poorly to ‘out-of-distribution’ data, where new data differs significantly from the initial training set (e.g. new field sites, different microphones). To address these issues, recent efforts have sought to develop broad multi-species classifiers trained on large and diverse libraries of recordings. Well-established pretrained bioacoustic networks have been primarily trained on bird taxa, where large open-source annotated libraries are available (e.g. Xeno-Canto (XC), Macaulay Library) [9,10]. These pretrained networks can sometimes be used off the shelf to classify sounds in new recordings, but this is restricted to only the classes present in their training set. Furthermore, these pretrained classifiers still underperform on novel datasets that are out-of-distribution from their training data and on classes that are under-represented in their training data [7,11], limiting their broad application and use.

When a pretrained network cannot directly classify novel signals, transfer learning represents an effective alternative. Here, samples from new target classes are passed through the pretrained network, and outputs from an intermediate layer are used to produce a feature embedding representation of the samples. These fixed embeddings can then be used to train a lightweight machine learning classifier [9,12]. This removes the costly training phase by using knowledge from the network’s embedding space learned during pretraining. Additionally, strong pretrained models facilitate few-shot transfer learning, where only a small number of training examples are needed to produce a highly accurate classifier, significantly reducing annotation costs [13]. Emerging research shows that networks pretrained on unrelated terrestrial bioacoustic data transfer well to similar bioacoustic domains, enabling few-shot learning [9]. However, the ability of pretrained bioacoustic networks to transfer to highly novel domains, such as aquatic environments, is largely untested [14]. Substantial domain shifts may require the development of novel pretrained networks to achieve accurate few-shot transfer learning. The optimal pretraining strategies to produce these networks remain unknown, which is further compounded by the sparsity of annotated libraries for novel domains.

Coral reef ecosystems host some of the highest documented bioacoustic diversity in the ocean [15,16], yet ML-accelerated PAM analysis is significantly underdeveloped for these habitats. Coral reefs host >25% of marine biodiversity, and >375 million people are directly reliant on the ecosystem services these habitats provide [17,18]. They are also among the most threatened habitats globally, with >50% of the world’s reefs lost over the last 70 years and a projected loss of 90% of those remaining by 2050 [19,20]. The soundscapes of these habitats have been found to contain information relevant to key ecosystem attributes such as coral cover and fish community diversity, as well as representing a key ecosystem function that drives larval recruitment [15,21]. PAM is therefore emerging as a promising tool to monitor these threatened habitats [22], but there is a sparsity of relevant annotated data. As is common for underwater soundscapes, the majority of biological sounds on coral reefs remain undocumented and for a significant portion of those that have been recorded, the taxonomic source of origin has not been validated [23,24]. Automated analysis of reef PAM data is therefore highly underdeveloped, with only a very limited number of studies having used ML-accelerated analysis on PAM data from these habitats [25–27]. Consequently, coral reef habitats represent an excellent candidate for assessing novel few-shot transfer learning bioacoustic frameworks, with advances in this field having the potential to help address real-world conservation challenges.

In this study, we aim to empirically identify an effective pretraining strategy to produce an efficient network that supports accurate few-shot transfer learning for a novel bioacoustic domain. Such a network should facilitate the analysis of PAM data with minimal computational and annotation costs. We selected the coral reef domain owing to the threatened status and high acoustic diversity of these ecosystems, with the potential to provide a strong foundation for transferring to other aquatic habitats. To achieve this, we first assembled ReefSet, the largest published dataset of annotated reef recordings to date, though it is only 1.99% the size of comparable bird libraries at the time of writing [28]. We then set out to determine how well existing pretrained networks perform at few-shot transfer learning on ReefSet. Next, we tested whether performance could be enhanced by (i) pretraining on this significantly smaller but in-domain dataset, and (ii) through cross-domain mixing during pretraining. Finally, to assess the generalizability of this strategy across the reef domain, we tested whether the output from cross-domain mixing optimized for the coral reef domain impacts generalizability to unrelated domains.

## 2. Methods

### (a) Data compilation

**Figure 1.**
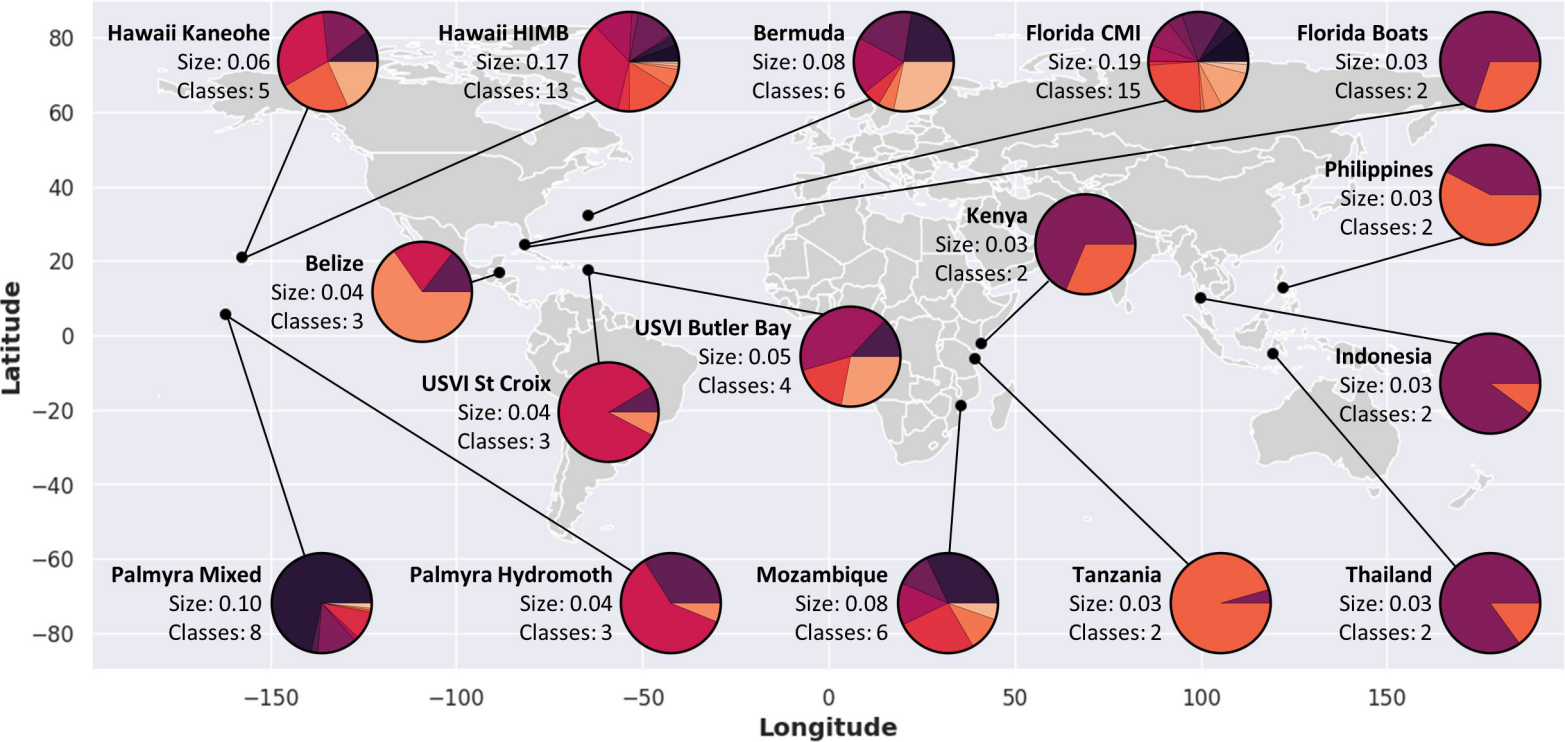
Datasets used to assemble ReefSet. Size indicates the relative size of each dataset to ReefSet, summing to one. Classes indicate the number of unique labels within each dataset. Pie charts indicate the distribution of labels within the dataset, with colours set independently for each dataset.

To maximize the generalizability of our approach, we compiled a diverse meta-dataset of 57 084 labelled coral reef bioacoustic recordings across 37 classes and from 16 individual datasets over 12 countries (figure 1; electronic supplementary material, S1, table S1), hereafter referred to as ‘ReefSet’. Each individual dataset was originally collected and labelled for different purposes, using a variety of sampling strategies and hydrophone models (electronic supplementary material, S1, table S2). During the annotation of each dataset, longer recording periods were segmented into samples of shorter windows (1.88 s) to fit within two window lengths of the industry standard networks YAMNet and VGGish (table 1) at the time of curation. Samples were labelled by human annotators using aural and visual inspection of each sample’s spectrogram. While many classes were of known origin, others were unknown but typically presumed to originate from fishes. All labelled samples were then re-sampled to 16 kHz and written out as separate waveform audio files.

**Table 1.** Details of the four pretrained networks used to evaluate transfer learning performance on ReefSet. (Real-time factor inference speed reflects how many times faster each network is at processing the audio’s real-time duration on a standard central processing unit (CPU); further details are in the electronic supplementary material, S1.)

network	training domain	number of training classes	input sample rate (kHz)	input length (s)	embedding dimension	parameter count (million)	real-time-factor inference speed (CPU)
VGGish	AudioSet (YouTube)	31 000	16	0.96	128	72.1	41.1
YAMNet	AudioSet (YouTube)	521	16	0.96	1024	4.7	86.04
BirdNET v2.3	bioacoustic (primarily birds)	3337	48	3	1024	10.4	260.68
Perch v1.4	bioacoustic (birds)	10 932	32	5	1280	80.1	39.41

To amalgamate class labels across datasets, each sample was first given a single primary label: biophony, anthrophony, geophony or ambient. Here, the ambient label was used for negative samples in the Florida-Boats, Kenyan and Indonesian datasets, where the annotation strategy used a positive label class (motorboat, fish noise and bomb fishing, respectively) alongside a strongly labelled negative class. A single secondary label was then applied to all other samples using existing labels from the datasets, with merging of labels under a common name where sounds matched across multiple datasets (e.g. motorboats). Within any one dataset, only samples containing one sound class were used. In some cases, co-occurrence of sound classes from other datasets may have been present in a sample, but this was minimized owing to the short sample length. Later, for evaluation, classifiers were trained on a maximum of 32 samples per class for each dataset, with a minimum of 10 samples from each class held out for testing. Therefore, classes with less than 42 samples in any given dataset were merged by applying only a primary label (biophony, geophony or anthrophony). Where the count of samples merged under the primary label class still did not total 42 or more samples in a given dataset, these samples were discarded. This yielded the final meta-dataset of 57 074 labelled samples, split across the four primary labels: biophony (79.20%), anthrophony (10.39%), geophony (0.09%) and ambient (10.32%), with 33 secondary labels (electronic supplementary material, S1, table S2).

### (b) Evaluating existing pretrained networks

We identified four pretrained networks widely adopted for use in acoustic transfer learning (table 1). All four networks employ a convolutional neural network (CNN) architecture. The first two were VGGish [29] and YAMNet [30], both trained on general-purpose audio datasets. VGGish was trained on the YouTube-70M dataset, a dataset consisting of 20 billion weak multi-label samples across 31 000 classes. YAMNet was trained on AudioSet, a large ontology of 2.1 million human-labelled acoustic events across 521 classes gathered from YouTube [31]. The second two were BirdNET [10] and Perch [9], both primarily trained on bird recordings from the XC repository. Perch was trained on the full corpus of XC bird recordings, split into 2.9 million samples, each 5 s in length, and was configured with hierarchical taxonomic output heads for species, genus, family and order classes. BirdNET was trained on a smaller set of bird classes than Perch overall but included bird samples from the Cornell Lab of Ornithology’s Macaulay Library [32], alongside 101 additional classes such as human speech, dogs and amphibian species.

For input to each network, audio samples were upsampled where required to match the input sample rate of the respective model (table 1). As samples were shorter than the input window size of BirdNET and Perch, zero-padding was applied to the tail end of each sample. As samples were twice the length of the input window size of VGGish and YAMNet, samples were split into two windows, feature embeddings were calculated for each window, and the mean across both was taken.

To evaluate the transfer learning capabilities of the four pretrained networks, for each of the 16 datasets in ReefSet, a pretrained network was configured with a final fully connected linear layer with output heads corresponding to the classes present in the respective dataset. This final layer was trained for 128 epochs using a batch size of 32, learning rate of 0.001 and categorical cross-entropy loss. This process was repeated using 4, 8, 16 and 32 training samples per class, with 10 repeats using a new random seed for the train-test split and initialization of each. For each seed, all remaining samples were set aside for testing, with a minimum of 10 per class. The mean area under the receiver operating characteristic curve (AUC-ROC) was calculated for the test set across the 10 repeats for each of the four training sample counts [33].

### (c) Pretraining with in-domain data

State-of-the-art bioacoustic few-shot learners commonly use CNN architectures [34]. We therefore adapted the pretraining protocol used for Perch, in which an EfficientNet CNN classifier is trained and used to extract high-quality feature embedding representations for transfer learning [9]. During training, datasets were filtered to remove any samples with the primary label ‘ambient’, in order to eliminate samples that may contain unintentional positive matches for classes in other datasets. For input to the network, samples were upsampled to 32 kHz and log-mel per-channel energy normalization (PCEN) spectrograms were calculated from each (electronic supplementary material, S1). Repeat padding was applied to samples, where the signal was repeated until they met the 5 s input shape. Samples were shuffled, and two augmentations were implemented throughout training: random normalization with a minimum and maximum gain of 0.15 and 0.25, respectively, and MixUp with a mix-in probability of 0.75 [35]. The Perch network architecture was adapted to be configured with hierarchical output heads for each primary label (biophony, geophony, anthrophony), with the 35 secondary labels nested within this minus any exclusive to held-out data. All training runs were completed for 200 000 steps.

For the first stage of the experiment, a hyperparameter sweep was performed in which models were trained on 14 of the 16 datasets, with two held out for validation (electronic supplementary material, S1). Learning rate, EfficientNet architecture and batch size were probed during the sweep. The core pretraining stage of the experiment was then performed using the optimum hyperparameters from the sweep (electronic supplementary material, S1, table S3).

To rigorously evaluate the performance of few-shot transfer learning on unseen datasets, we used a leave-one-dataset-out (LODO) approach. During LODO, pretraining was first undertaken using 15 datasets from ReefSet, maintaining one held-out dataset for evaluation. This was repeated in all combinations one by one to produce 16 pretrained models. In the second stage of LODO, evaluation of each model was performed on its respective held-out dataset following the same few-shot transfer learning protocol as described for the existing pretrained networks. Given all data originated from reef habitats, the evaluation data could be considered in-domain while being out-of-distribution.

### (d) Pretraining with cross-domain mixing

**Figure 2.**
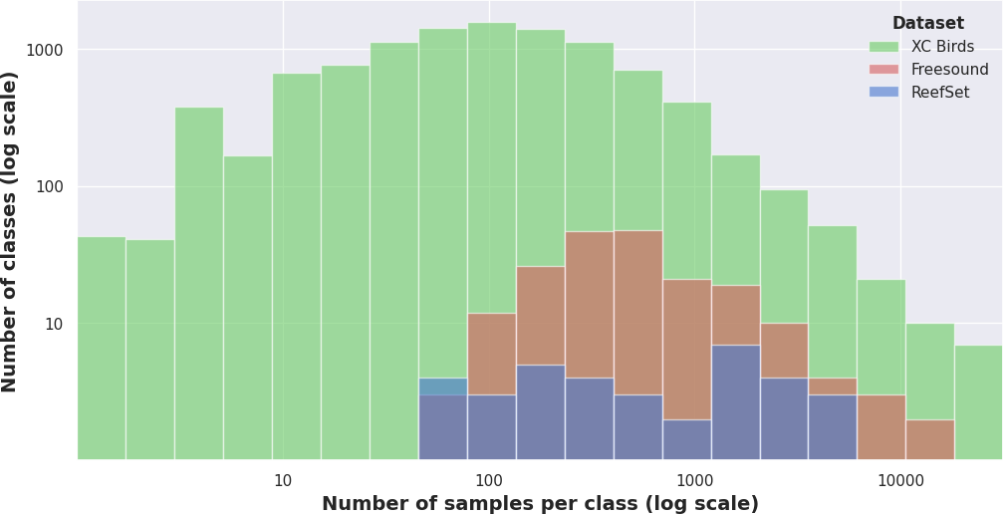
Histogram of counts by class for the three datasets used in pretraining: XC Birds, Freesound and ReefSet. Bins are logarithmically spaced based on the range of counts in the XC Bird data, with bin edges determined by creating 20 equal intervals on a logarithmic scale between the minimum and maximum counts observed in the XC Bird dataset.

We first tested mixing the full XC Bird catalogue of 2.9 million samples used to train Perch with the more modest ReefSet, approximately 1.99% of the size in total sample count (figure 2). The XC Bird dataset was used without modifications to the original pretraining of the Perch model [9]. This mixing provided a total of 10 165 target classes, minus any exclusive to held-out reef data for LODO evaluation. To integrate the XC Bird dataset into the training procedure, the model was configured with the same output heads for ReefSet, alongside additional species, genus, family and order output heads for the XC Bird dataset, with a loss weighting of 0.1 for the latter three compared to standard heads.

Next, alongside ReefSet and the XC Bird dataset, we mixed in Freesound Dataset 50K [36], a dataset based on the AudioSet ontology consisting of 108.2 h of annotated sound events across 200 classes (figure 2). Freesound includes audio from a more general selection of sound events, comparable to the domain used to train VGGish and YAMNet, but with fully open-source access to the audio whereas AudioSet must be scraped from YouTube. Our only adjustment to the Freesound dataset was to remove all samples with the label ‘bird’ (3.38% of the dataset) to mitigate overlap with more taxonomically detailed labels in the XC Bird dataset. The network was then configured following the XC Bird cross-domain mixing strategy, alongside an additional set of output heads for Freesound for a total of 10 364 target classes.

During pretraining for both domain mixing strategy experiments, individual datasets were cycled back in once all samples from them had been used once. As with the ReefSet pretraining strategy, a hyperparameter sweep was performed, with an additional parameter for dataset weighting (electronic supplementary material, S1). All other components remained unchanged, and the LODO approach was used for pretraining and evaluation.

### (e) Evaluating SurfPerch on novel bioacoustic domains

Using SurfPerch, the resultant network after optimizing the highest performing strategy in our pretraining experiments, we mirrored the evaluation protocol used in the study by Ghani *et al*. [9] to assess the ability of Perch to generalize to novel bioacoustic domains. These domains originated from the recordings of birds, frogs, bats and marine mammals. Sample counts ranging from 4 to 256 training samples per class were used, with 10 repeats of each. SurfPerch was evaluated in an ‘off the shelf’ manner, with no hyperparameter sweeps or pretraining used to optimize for the novel bioacoustic domains being tested. These novel datasets originated from the bird, bat, frog and marine mammal domains; see Ghani *et al*. [9] for further details on the data. As with the other pretraining experiments, a new network was configured with a final classification head to match the target classes for each respective dataset, which was then fine-tuned while the rest of the weights were kept frozen. Fine-tuning was conducted for 128 epochs using a batch size of 32, a learning rate of 0.001 and categorical cross-entropy loss. The fine-tuned networks were then evaluated on held-out test sets from their respective datasets. Fine-tuning was performed across multiple counts of training samples per class for each dataset, ranging from 4 to 256, with 10 repeats for each count using a new random seed to select the training data.

## 3. Results

### (a) Pretrained bioacoustic networks outperform networks pretrained on general audio

**Figure 3.**
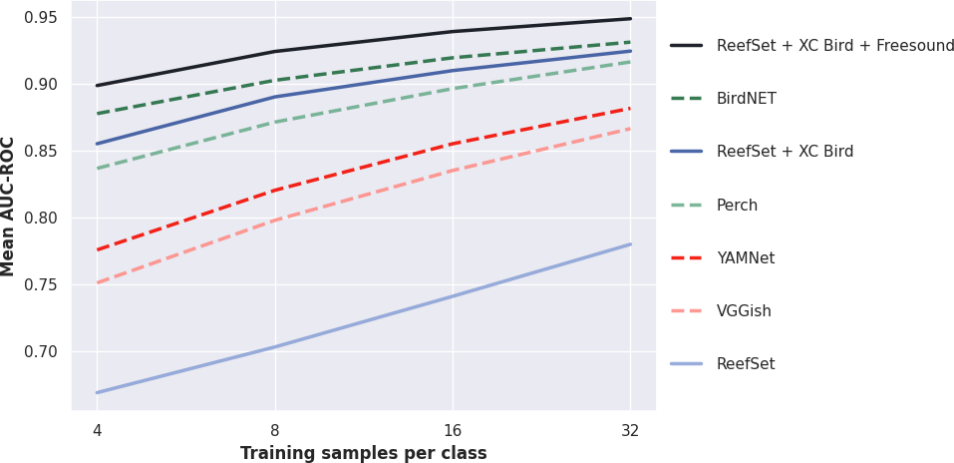
Mean AUC-ROC scores reported by transfer learning evaluation for each model across all 16 datasets within ReefSet. Dashed lines represent existing pretrained networks, with names indicated in the legend. Solid lines represent the three alternative pretraining strategies for our model, with the data used for pretraining indicated in the legend.

Mean AUC-ROC scores revealed that the pretrained networks ranked consistently across all four training sample counts. In ascending order, the means and standard deviations of these were as follows: BirdNET (0.908 ± 0.09), Perch (0.881 ± 0.11), YAMNet (0.834 ± 0.05) and VGGish (0.813 ± 0.05) (figure 3). These results revealed that the two networks pretrained primarily on the bird domain outperformed the two trained on more general YouTube data. As expected, the mean AUC-ROC scores of all pretrained models improved, and the standard deviation decreased, as the number of training samples per class increased from 4 to 32 (figure 3; electronic supplementary material, figure S1).

**Figure 4.**
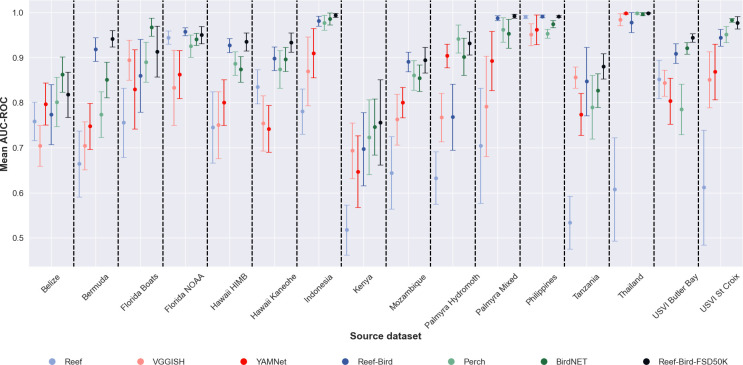
Mean AUC-ROC scores reported by transfer learning evaluation on each dataset within ReefSet across all training samples per class counts used (4, 8, 16 and 32). Points represent the mean, and lines represent the standard deviation. Within each dataset bin, models are ordered by overall mean performance across all dataset and training sample counts, going from weakest (left) to strongest (right). In the legend, existing pretrained networks are indicated by name, whereas our alternative pretraining strategies are indicated by the datasets used during pretraining.

Considering the constituent datasets within ReefSet on an individual basis, these presented a range of apparent difficulty and complexity (figure 4; electronic supplementary material, figure S1). The datasets from Thailand, the Philippines and Indonesia, which only required binary classification between one anthropogenic and one biophonic class, generally presented easier challenges with mean AUC-ROC scores of 0.994 (±0.007), 0.960 (±0.010) and 0.935 (±0.056), respectively, across all four pretrained networks and sample counts. More challenging datasets were those which required the prediction of multiple classes, where samples were labelled with secondary biophony labels alongside samples labelled only with the primary biophony label class. These more challenging tasks included the Kenya, Belize and Tanzania datasets, with mean AUC-ROC scores of 0.703 (±0.043), 0.791 (±0.065) and 0.812 (±0.037), respectively, across all four pretrained networks and sample counts. Using just four training examples per class with BirdNET, the overall best performing pretrained network, the lowest and highest AUC-ROC scores were reported for the Kenya and Thailand datasets, with mean AUC-ROC scores of 0.746 (±0.062) and 0.996 (±0.006), respectively, across the 10 random seeds used for each.

### (b) Existing pretrained networks outperform pretraining on limited in-domain data

Our second experiment revealed that the few-shot transfer learning capabilities of a CNN pretrained on our highly in-domain but smaller ReefSet meta-dataset were considerably lower than those of all four existing pretrained networks. This strategy reported a mean AUC-ROC score of 0.724 (±0.05) across all four training sample counts, lower than any of the pretrained networks. The ReefSet-only pretraining strategy had a 200.72% and 47.80% higher AUC-ROC error (area above the ROC) than BirdNET and VGGish, the highest and lowest performing pretrained networks, respectively.

### (c) Cross-domain pretraining improves generalizability

Our third experiment revealed that cross-domain mixing of the small in-domain ReefSet dataset with a large set of out-of-domain bird bioacoustic data provided considerable improvements. Using the LODO pretraining and evaluation procedure once again, we observed a mean AUC-ROC score of 0.895 (±0.03) across all four training sample counts using this cross-domain pretraining strategy (figure 3). This represented a notable improvement over pretraining on the in-domain ReefSet alone, which had a 163.90% higher AUC-ROC error. Importantly, this also achieved a 12.32% improvement in AUC-ROC error upon pretraining with the XC Bird dataset alone, represented by the pretrained Perch model. The only pretrained network that still outperformed this cross-domain pretraining strategy was BirdNET, with our ReefSet and XC Bird cross-domain pretraining strategy having a 12.24% higher mean AUC-ROC error.

Our fourth experiment revealed that expanding the diversity of data used in cross-domain pretraining further enhanced few-shot transfer learning capabilities on the novel coral reef domain. Using the LODO train and evaluation protocol, this triple-domain pretraining achieved the highest mean AUC-ROC scores of any strategy, with a mean AUC-ROC of 0.928 (±0.02) across all four training sample counts (figure 3). This represented a 31.26% reduction in error compared to cross-domain pretraining with the ReefSet and XC Bird datasets. Importantly, this strategy also outperformed BirdNET, the previously highest scoring network, which had a 21.68% higher AUC-ROC error. Using just four training samples per class, this triple-domain pretraining strategy achieved a mean AUC-ROC of 0.900 (±0.02) across the 16 datasets.

Final trials using the triple-domain strategy revealed modifications to the bias, gain and smoothing parameters of the PCEN spectrogram (electronic supplementary material, S1), and pretraining for 1 m steps further improved performance. Following the LODO pretraining and evaluation protocol, a mean AUC-ROC score of 0.933 (±0.02) was reported using these adjustments, representing the strongest overall performance. This improvement corresponded to 26.84 and 6.60% mean improvements upon the AUC-ROC error of BirdNET and our initial triple-domain pretraining trial, respectively (electronic supplementary material, S1, figure S2). Finally, we produced SurfPerch, the open-source version of this model (electronic supplementary material, S2), by pretraining with this triple-domain strategy, including the full ReefSet meta-dataset, using all 16 source datasets.

### (d) Targeted cross-domain pretraining does not improve generalizability to non-target bioacoustic domains

**Figure 5.**
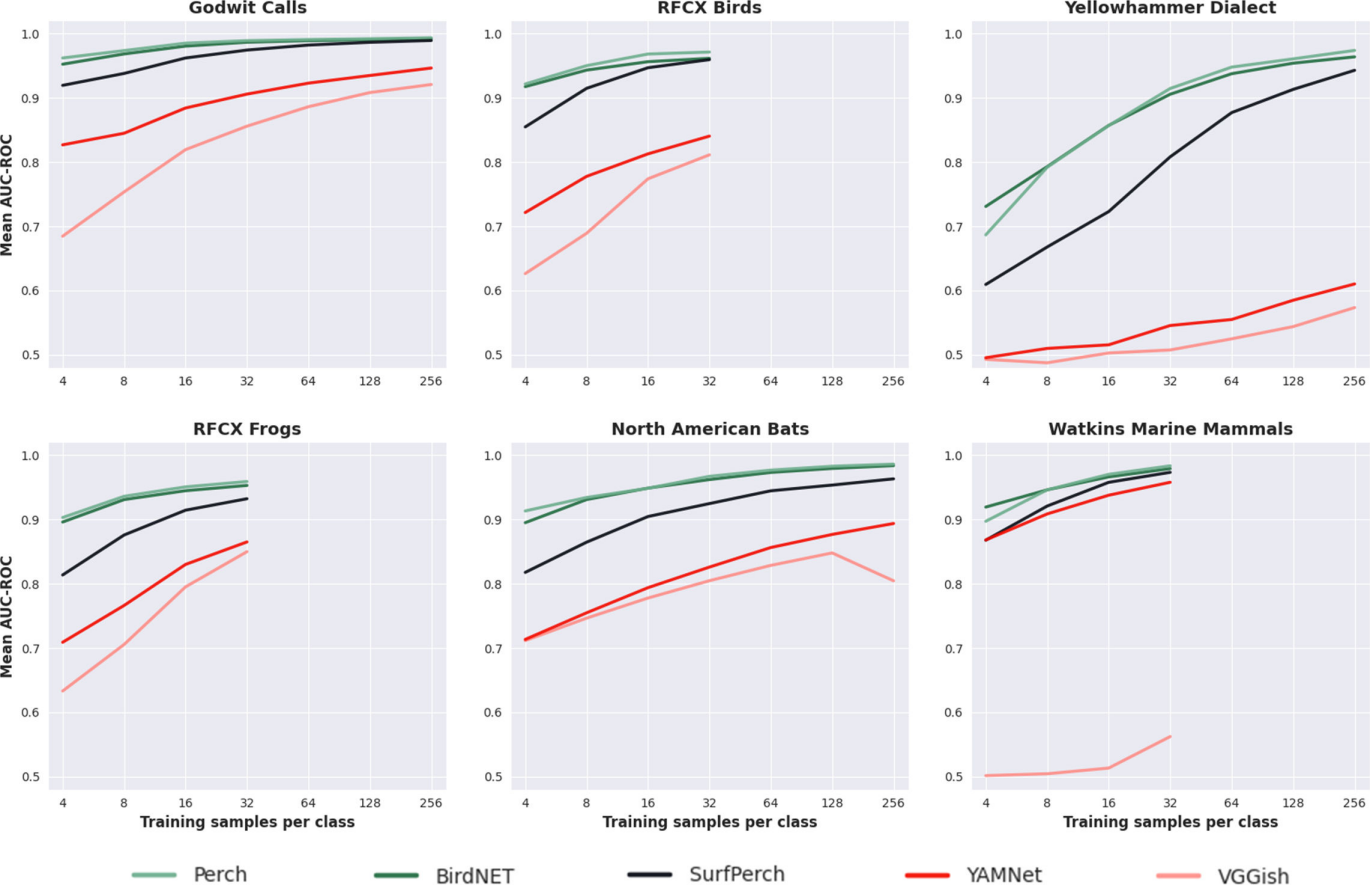
Mean AUC-ROC scores reported from transfer learning evaluation of each model on six novel bioacoustic datasets. Each model and training sample per class count combination was repeated across 10 random seeds.

While cross-domain mixing reported notable generalizability improvements to the reef domain, we observed this strategy negatively impacted performance on alternative bioacoustic domains that were not optimized for during pretraining (figure 5). We observed that Perch and BirdNET outperformed SurfPerch in all six of the novel domains. The lowest performance gap between SurfPerch and Perch, the overall best performing pretrained network at the novel challenges, was observed for the Godwit Calls and Watkins Marine Mammals datasets, with mean AUC-ROC scores 0.019 lower than Perch for both datasets across all training sample counts per class. The largest performance gap between SurfPerch and Perch was observed for the Yellowhammer dialect dataset, with a mean AUC-ROC score 0.084 lower than Perch across all training sample counts per class. However, SurfPerch did outperform both YAMNet and VGGish across all datasets. As the training samples per class counts increased, the performance gap between SurfPerch and Perch decreased for each, with a difference between mean AUC-ROC scores across all datasets of 0.067 for four training samples per class and 0.012 for the maximum training sample count per class (32 or 256).

## 4. Discussion

We show that by using multiple domains during pretraining, we can achieve far superior transfer learning capabilities for the data-deficient domain of marine bioacoustics. In doing so, we present a novel pipeline that could resolve the considerable bottlenecks in the analysis of tropical reef and similar acoustic domains. We began by testing the transfer learning capabilities of existing pretrained networks on marine bioacoustic data. We found that networks pretrained on data from bioacoustic domains outperformed those pretrained with more general audio data from unrelated domains. Next, we show that these existing pretrained networks outperformed pretraining with our highly in-domain, but smaller, ReefSet meta-dataset. We then found that cross-domain mixing, using the larger out-of-domain XC Bird dataset with the smaller in-domain ReefSet improved upon the transfer learning capabilities of the previous strategies. Finally, we reveal that mixing together all three domains during pretraining provides the strongest performance. Importantly, however, we observe that cross-domain mixing did not improve performance on unrelated bioacoustic domains that were not optimized for during pretraining. These findings present a powerful strategy to produce pretrained networks for bioacoustic and other acoustic domains that do not currently have adequate pretrained networks for use in transfer learning.

The product of our optimum pretraining strategy for marine bioacoustic data, SurfPerch, supports accurate few-shot learning. Furthermore, this was evaluated by fine-tuning only the final layer of the network during transfer learning. This combined few-shot transfer learning protocol therefore significantly reduces both the annotation and computational costs required to build accurate classifiers, enabling end-users to perform this on standard personal computing devices using a significantly reduced set of annotations. Example uses of tropical reef bioacoustic analyses that could be accelerated or scaled using SurfPerch include reef health assessments [37], tracking reef restoration success [5], measuring marine protected area outcomes [38] or understanding fundamental processes within the soundscape such as temporal patterns and biogeographical variability [39,40]. Given its potential applications, we have provided an interactive demonstration on how to implement this approach on new data entirely from a web browser (electronic supplementary material, S2), including the incorporation of an agile modelling protocol that can be used to further boost classifier performance by identifying the most relevant samples for annotation [41]. A simplified workflow for fine-tuning on new data is detailed in the electronic supplementary material, figure S3.

The results we present here provide key advancements in pretraining for bioacoustics. The performance of existing pretrained networks supports similar work by Ghani *et al*. [9], which reported that networks pretrained on large and diverse bird bioacoustic datasets generalize better to other bioacoustic domains than those pretrained on more general audio. The reasons behind this remain an open research question. This could be owing to common properties between bioacoustic domains, or the high innate acoustic complexity and variety of bird vocalizations compared to AudioSet. The increased volume of training data probably also contributed to the improved performance of our two- and three-way cross-domain mixing strategies. However, class diversity has been empirically demonstrated as a more significant driver of generalization in multiple settings [42–44]. Both bioacoustic networks were pretrained on a far larger diversity of classes compared to YAMNet, whereas VGGish was trained on the largest diversity of classes, but these were weakly labelled (table 1). While BirdNET was trained on a lower class diversity, its outperformance of Perch provides potential evidence that cross-domain mixing during pretraining is also a key factor in enhancing generalizability. BirdNET’s (v2.3) pretraining included invertebrate, amphibian, mammal and anthropogenic sound classes. Future experiments controlling for data volume and domain diversity across datasets could help disentangle the contributions of class and domain diversity versus data quantity further. Lastly, given that zero-padding was used to lengthen ReefSet samples during transfer learning to match the input length of Perch and BirdNET, the latter’s shorter fixed window length may have been favourable owing to reduced padding. Where padding is necessary owing to cross-domain mixing, future experiments could compare models using repeat-padding versus zero-padding.

Our findings present guidance for future users aiming to use deep neural networks for bioacoustics. Pretraining experiments such as those presented here are inherently computationally expensive. The experiments outlined total to the training of 139 networks, on average requiring approximately 20 h on a TPUv3 pod, the equivalent of 26 668 USD using Google Cloud spot instances at the time of writing. By comparison, fine-tuning a model is arbitrary, taking less than 1 min on a CPU with 128 training samples using a standard personal laptop. Bioacousticians with novel datasets will therefore benefit most from identifying an existing pretrained network most relevant to their domain or from testing a suite of existing networks, as presented in our first experiment. Here, we show that SurfPerch presents the strongest option for coral reefs and probably for related aquatic domains, whereas Perch, trained exclusively on bird data, presents a better option for unrelated bioacoustic domains. Interestingly, Perch still outperformed SurfPerch on the Watkins Marine Mammal dataset. Many of the sounds in the Watkins dataset were in fact terrestrial marine mammal vocalizations, which may partially explain this [45]. Additionally, marine mammal calls typically consist of multiple high-frequency phonemes [46], potentially making them more comparable to the bird domain than the single-phenome sounds characteristic of most fish vocalizations (e.g. grunts, pops).

Where an adequate pretrained network is not available and domain-specific data is sparse, we show that cross-domain pretraining presents a valuable strategy to develop a suitable network for the target domain. Future work may be able to improve upon the strategies tested here. Increasing the volume of high-quality in-domain training data is typically the most valuable tool for improving model performance [47]. Relevant sound libraries for the coral reef domain with potential for growth include the FishSounds platform [48] and the proposed Global Library of Underwater Biological Sounds [23]. Furthermore, growing open-source sound event libraries are available, meaning additional bioacoustic and unrelated acoustic domains could be integrated [49–51]. Ongoing efforts to release updated versions of existing bioacoustic networks, which incorporate an increased diversity of data from both bioacoustic and other domains into pretraining, will probably improve their generalizability to novel bioacoustic domains. Indeed, updated versions such as BirdNET v2.4, which has been trained on over twice the number of classes, could be used to validate this. More broadly, mixing data from a diverse set of domains and optimizing for a range of them during evaluation, rather than the single domain we targeted here, may present a route to developing improved foundational bioacoustic and acoustic models for wider contexts. While we restricted our pretraining in the present work to fully open-source datasets, incorporating AudioSet in its entirety, with its greater data volume and class diversity, could provide a direct route to improving generalizability during transfer learning for bioacoustic models. Additionally, including bioacoustic data in the pretraining of industry-standard models may offer reciprocal improvements in their generalizability.

Beyond enhancing the data used for training, future work could also explore methodological improvements. Marine PAM data is inherently noisy, with recordings typically comprising multiple sources from biophony, geophony and anthropogenic components [22]. Implementing an unsupervised source separation model presents a proven tool for improving classification in noisy bioacoustic datasets by disentangling individual signals from the broader soundscape [52,53]. Elsewhere, self-supervised learning (SSL) presents a tool that can be used to learn informative features from unlabelled data, enabling it to exploit vast unannotated datasets [54]. Future work could benchmark pretraining on larger in-domain datasets with SSL against cross-domain pretraining of supervised classifiers. Other changes during the transfer learning component could boost performance. First, alternative lightweight classifiers (e.g. two-layer models, random forests) could be tested. Augmentations are another proven way to improve classification accuracy with limited training data [7]. Better still, agile modelling, alternatively known as active learning, can be integrated into the pipeline, using a human-in-the-loop approach to identify the most informative training samples [41,55].

In conclusion, we used cross-domain pretraining to develop a powerful tool that supports automated analysis of marine PAM recordings with low annotation and computational costs for the end-user. Our findings offer insights into replicating this for other acoustic domains where existing pretrained networks are inadequate. Combining efficient machine learning analysis such as ours with the vast scales at which PAM data can be collected has significant potential to boost our understanding and monitoring capacity of global biodiversity. We anticipate that these technologies will facilitate the expansion of scientific frontiers towards new applications and challenges so far unrealized.

## Data Availability

The pretrained SurfPerch model is available at: https://www.kaggle.com/models/google/surfperch. The pretrained SurfPerch model, alongside the full ReefSet dataset presented in this study are also available in the Zenodo repository [56]. A tutorial on using SurfPerch from a web browser is available on GitHub: https://github.com/BenUCL/surfperch/blob/surfperch/SurfPerch_Demo_with_Calling_in_Our_Corals.ipynb. Supplementary material is available online [57].
